# Supporting people with chronic kidney disease to self-manage their condition: understanding the lived experiences, needs and requirements, and barriers and facilitators

**DOI:** 10.1007/s40620-025-02349-8

**Published:** 2025-07-12

**Authors:** Courtney J. Lightfoot, Thomas J. Wilkinson, Matthew P. M. Graham-Brown, Alice C. Smith

**Affiliations:** 1 Leicester Kidney Lifestyle Team, Department of Population Health Sciences, University of Leicester, Leicester, UK; 2 NIHR Leicester Biomedical Research Centre, Leicester, UK; 3 Diabetes Research Centre, University of Leicester, Leicester, UK; 4 Department of Cardiovascular Sciences, University of Leicester, Leicester, UK; 5 Department of Renal Medicine, University Hospitals of Leicester NHS Trust, Leicester, UK

**Keywords:** Chronic kidney disease, Health behaviour, Patient centred-care, Self-care, Self-management

## Abstract

**Background:**

Self-management has been identified as an essential component in the effective management of patients with chronic kidney disease (CKD). To effectively develop interventions that support patients with CKD to self-manage, it is crucial to understand their experiences and the factors that may influence their ability to self-manage. This study explored awareness, attitudes and participation with self-management in people living with non-dialysis CKD to understand factors influencing self-management behaviours.

**Methods:**

Semi-structured interviews were conducted with 22 individuals living with non-dialysis CKD. Topics explored included perspectives and experiences of self-management, health and lifestyle behaviours, healthcare professional support, and self-management support, including future interventional approaches. Data were audio recorded and transcribed verbatim. Thematic analysis was used to analyse the data and to identify and report themes.

**Results:**

Six themes were identified encompassing perspectives, barriers and facilitators of self-management: “perceptions and experiences of (self-)managing CKD”, “perceived needs and requirements for self-management education and support”, “knowledge and capability-related factors”, “skills and opportunity-related factors”, “confidence and motivational-related factors” and “social support”.

**Conclusion:**

Participants perceived that their CKD was not a significant problem, given the lack of concern from their doctor. Despite reporting a lack of awareness and understanding of CKD and its management, participants expressed interest in learning more and implementing appropriate self-management strategies. It was perceived that information and support were provided when it was almost too late, and not when it could potentially have the greatest impact. Perceived barriers and facilitators must be considered when developing interventions to support self-management for people with CKD.

**Graphical abstract:**

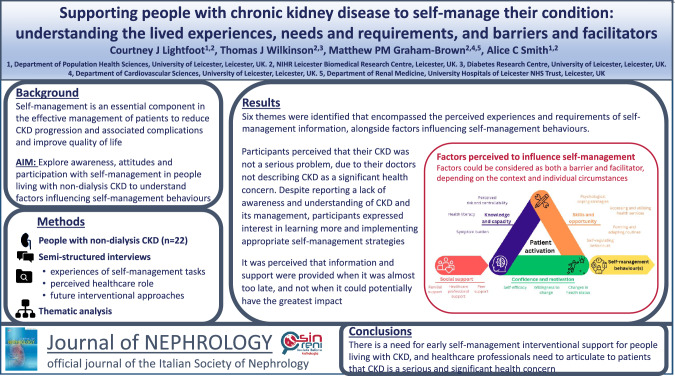

**Supplementary Information:**

The online version contains supplementary material available at 10.1007/s40620-025-02349-8.

## Introduction

Chronic kidney disease (CKD) is a long-term condition associated with high levels of morbidity and mortality [1]. CKD is usually insidious, and individuals are often asymptomatic until the disease becomes advanced [1]. Living with CKD can negatively impact an individual’s quality of life (QoL) and is independently associated with adverse health outcomes and consumption of healthcare resources [2]. Most individuals living with earlier stages of CKD are managed in primary care with little or no input from secondary care kidney specialists and no resources to effectively support self-management. Self-management has been identified as a key part of the National Health Service England long-term strategy to improve care and outcomes for patients with long-term conditions [3] and for National Institute for Clinical Excellence guidelines for CKD management to develop strategies to improve CKD self-management [4]. The goals of self-management programmes are to help individuals take responsibility for all, or some, aspects of the day-to-day management of their condition [5], by engaging in health-promoting behaviours, such as medication adherence, health monitoring, symptom monitoring, and lifestyle modifications (e.g., physical activity and diet) [6, 7]. For people with CKD, these behaviours slow progression of the disease, reduce the risks of associated complications [6, 8], and improve QoL [8].

To effectively manage one’s own health, an individual needs to have the appropriate knowledge, skills, and confidence—termed patient activation [9]. As patient activation is a dynamic behavioural concept, the degree to which an individual engages in health-promoting behaviours that promote successful self-management changes over time [10]; improvements may be observed when an individual’s readiness to change improves and/or appropriate support is provided [10], whilst declines may result from worsening health status and perceived lack of energy and/or time to engage in illness-related activities [11].

Understanding the barriers and facilitators to self-management in people living with CKD is gaining interest to support the development of interventions. Thus far, research has focused on patients receiving kidney replacement therapies, with little research exploring self-management experiences of individuals with earlier-stage CKD. Improving our understanding of the self-management behaviours in patients with earlier-stage CKD is an important part of the strategy to reduce the risk of patients progressing to kidney failure, needing kidney replacement therapies, and suffering the adverse health risks associated with CKD. To do this, we must first understand the barriers and facilitators to effective self-management in this population.

This study explored awareness, attitudes, and participation towards self-management in people living with non-dialysis CKD to understand factors influencing self-management behaviours and how these may be targeted in a UK population.

## Methods

### Study design

This qualitative sub-study forms part of the mixed-methods study DIMENSION-KD (ISRCTN84422148) which comprised surveys, qualitative interviews, and physiological outcomes. Qualitative interviews were conducted to explore in-depth accounts of patients’ experiences of living with CKD and the impact this has on their life, lifestyles, and QoL. Data presented here focus on patient perceptions, experiences, and attitudes towards barriers and facilitators to CKD self-management.

### Participants

Participants were recruited from routine outpatient clinics at the Leicester General Hospital (LGH) or via the survey section of the study. Those already enrolled in the study (via survey sections) had the option to take part in an interview, and included participants from outpatient clinics at LGH (secondary care), or local general practitioner (GP) practices within Leicestershire (primary care), UK. Inclusion criteria were ≥ 18 years of age, CKD 1–5 not requiring kidney replacement therapy, and the ability to provide written informed consent. Exclusion criteria were receiving kidney replacement therapy (dialysis or kidney transplantation). Participants were contacted, via phone or email, to arrange a suitable date and time for their interview.

### Sampling

The study aimed to recruit 20 participants. This sample size was chosen based on information power [S1], and was believed to reflect sufficient diversity in views and experiences within the available time and resources; however, it was subject to change based on the richness of the data derived from participants. To capture diversity, participants were recruited using purposive sampling, utilising maximum variation sampling to ensure a representative population with range of characteristics. Given the relatively narrow focus of the study, combined with the purposive sampling of participants, perceived robust dialogue during the interviews based on the researcher’s experience, and diversity of participant’s perspectives, suggested that the proposed sample size would generate sufficient information power. The adequacy of the sample size was continuously reviewed during data collection to ensure an in-depth understanding of the investigated phenomena was obtained.

### Topic guide development

An interview topic guide was iteratively developed through familiarisation with current literature and exploring the three self-management tasks described by Corbin and Strauss [S2], alongside input from a patient and public involvement group on their main priorities and needs around self-management. The interview topic guide was comprised of two parts. Part one focused on experiences of living with CKD and the impact CKD has on an individual’s ability to self-manage, while part two focused on participant's perceptions and attitudes towards self-management support. The topic guide comprised topics including, but not limited to, symptoms, psychological impact, self-management, lifestyle, healthcare professional support, and perceptions of self-management support, with probes and prompts for each topic. The topic guide was piloted with two individuals with non-dialysis CKD to ensure that it was appropriate, easily understood, and functional. Additional questions and probes were added to the topic guide to allow for a more in-depth interview. As the pilot interviews were data-rich, they were subsequently included in the final analysis.

### Interview procedure

Individual semi-structured interviews were conducted face-to-face in a private room at LGH, by an experienced qualitative researcher (CJL). Participants had no relationship with the researcher prior to study commencement. The researcher kept a personal reflective logbook during interview conduction and analysis. Interviews were audio-recorded and transcribed verbatim by a professional transcription service.

### Ethics

Ethical approval was provided by the East-Midlands Leicester Central Research Ethics Committee (18/EM/0117). Written informed consent was obtained prior to the commencement of the interview. Transcripts were anonymised and IDs were given to participants. To ensure appropriate methodological practices and quality assurance, the eight ‘Big-Tent’ criteria [S3] were applied and the work conducted was assessed against the criteria.

### Data analysis

QSR International’s NVivo12 software was used to manage the data, which were analysed using reflexive thematic analysis, described by Braun and Clarke [S4]. The researchers familiarised themselves with the complete data set by listening to audio-recordings and annotating transcripts, and independently identified initial codes using an inductive approach. Potential themes were created through identifying relationships between codes, refocusing and collating them to form over-arching concepts. The researchers refined themes, and definitions of themes were agreed. The COREQ guidelines were followed when reporting the methods.

### Reflexivity statement

The first author (CJL) is an experienced mixed-methods researcher with expertise in patient experiences of living with and self-managing their long-term condition(s), particularly CKD. TJW is an experienced researcher in patient outcomes in multiple long-term conditions, notably CKD. MPMGB is a Consultant Nephrologist and an Associate Professor of Renal Medicine. ACS is a Professor of Lifestyle Medicine and has been conducting kidney research for > 35 years. The authors are aware of their own experiences of working with people with CKD, and how these may shape their views and understanding of CKD self-management, which may impact the data analysis. The lead author actively engaged in reflexivity and kept a personal reflective logbook to document their thoughts, feelings, and experiences throughout the research process to help identify potential biases and mitigate their impact. The authors engaged as a research team to ensure interpretations of the data were consistent and transparent and to reduce researcher bias.

## Findings

### Participant characteristics

A total of 22 participants (*n* = 11 males), mean age 71.4 years (range 48–88 years), mean eGFR: 42.2 (± 13.9) ml/min/1.73 m^2^) living with non-dialysis CKD were recruited to this qualitative sub-study, and interviewed. Of these, 14 (*n* = 64%) were recruited from primary care sites. The majority of participants were White British (*n* = 20, 91%). Participant characteristics are displayed in Table 1. Interviews lasted an average of 57 min (range 28–99).

**Table 1:** Participant characteristics

Participant ID	Gender	Age	Ethnicity	Education	eGFR (ml/min)
SC_01	Female	78	White British	University	33
SC_02	Male	78	Indian	University	29
SC_03	Male	78	White British	High school	19
SC_04	Female	48	White British	College	60
SC_05	Male	69	White British	High school	20
SC_06	Male	64	White British	Other Trade/vocation qualification	45
SC_07	Male	65	Pakistani	University	48
SC_08	Female	60	White British	University	16
PC_01	Female	67	White British	Other Trade/vocation qualification	47
PC_02	Female	65	White British	University	39
PC_03	Male	72	White British	University	47
PC_04	Female	74	White British	High school	57
PC_05	Male	64	White British	College	38
PC_06	Male	80	White British	Not reported	Unknown
PC_07	Male	77	White British	University	52
PC_08	Female	81	White British	None	40
PC_09	Female	57	White British	High school	62
PC_10	Male	68	White British	High school	62
PC_11	Female	88	White British	None	30
PC_12	Male	80	White British	College	57
PC_13	Female	86	White British	college	Unknown
PC_14	Female	71	White British	High School	44

*SC* secondary care, *PC* primary care

### Overview of findings

**Fig. 1:**
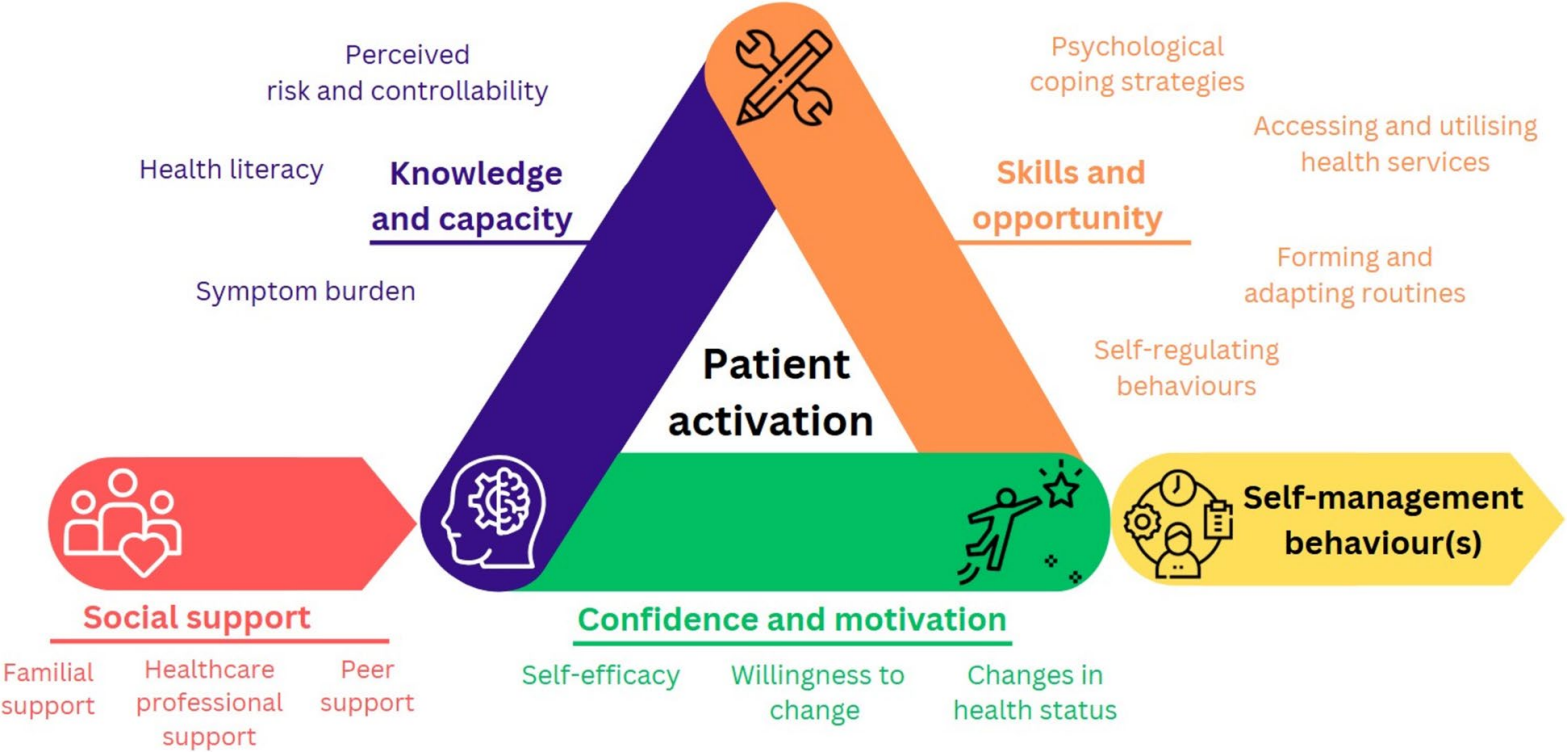
Schematic displaying factors perceived to influence self-management. Factors could be considered as both a barrier and facilitator, depending on the context and individual circumstances

Six overarching themes were identified, each containing subthemes (Tables 2 and 3). Many of the identified factors perceived to influence self-management, which were identified and categorised into four overarching themes, could be considered as both a barrier and facilitator, depending on the context and individual circumstances; these factors are based on constructs of patient activation [12] and the COM-B model [13], and are presented in a schematic displayed in Fig. 1.

**Table 2:** Themes and subthemes relating to lived experiences of CKD and self-management

Perceptions and experiences of (self-)managing CKD	
*Lack of awareness and understanding of CKD and its self-management*	“Once I had it, I had it; can’t do much about it. And I did ask, is it reversible? I think they said no, I don’t think it is reversible … I don’t understand how I got it … no-one’s ever told me how I got this disease. I’ve never really actually tried to work out” *SC_07* “I wasn’t aware that I’d got it. I hadn’t got any symptoms at all … no knowledge of having impaired renal function at all … I didn’t realise that it was as significant as it is … I think it’s just a matter of is there anything else that I need to know about to be able to improve my management of impaired renal function” *PC_01* “I don’t know whether it would be any medication required or involved or anything … I don’t know whether, however bad my condition is, good or bad it is, whether it deteriorates, I don’t know. Or will it just stay the same? No idea” *PC_10*
*A paucity of information received*	“I’m not aware you can do a lot for kidney. But I might be surprised … nobody’s probably turned around and said you’ve got to do this, this and this, because it is a condition that you’ve got, and it’s going to get worse. They’ve not really told me that” *PC_05* “No one has explained it other than they’re inefficient, put me on these tablets, and I never heard no more about it. No one has ever sat down and explained … why it is I’m having to take them. And how long I’ve got to take them for. Is it the rest of my life, or is it just 12 month?” *PC_06* “I’m a bit in the dark about that to be honest. As I say, they haven’t given me any information at all” *PC_12*
*Perceived downplay of kidney disease*	“Ignorance is bliss. If somebody said to me the symptom is so and so, I’d think oh my god, I’ve got it. So no, I don’t want to know … Can’t be that bad, otherwise the doctors would have said something to me. So they’re not bothered, I’m not bothered” *PC_14* “When [Doctor] said to me about kidney function was reduced, that I had got chronic kidney disease, I was a bit surprised. I must have said to him how significant is that, and I’ve got a feeling he didn’t really sort of see it as a big thing … I was led to believe that it wasn’t a major problem … they seem to play it down as not being a big issue” *PC_01* “I’ve never seen it as a big problem until now. The doctors have never been that worried … he says, isn’t what it should be on a normal person, but it’s nothing to worry about as regards you’re going to start to notice it yourself as such. Or it’s going to have an impact on your life” *PC_05*
*Engagement with self-management behaviours*	“I don’t think it’s necessarily changed my outlook or my condition, my general health. I still try to do things as it were, even if the kidney’s there, kidney condition, because obviously, as I say, you’ve got to live with it” *SC_05* “I have been asked to drink a little less, and probably eat a little healthier … I’ve not taken them on board if truth be told. Probably because I enjoy the lifestyle I have” *PC_10* “Looking back on life, that’s probably what’s led to diabetes, because I’ve ate what I want, as opposed to eating what I should eat … I’m quite happy what I’m doing at the moment to be honest. I supposed everybody could do a bit more, but I wouldn’t feel like [it]” *PC_05* “I enjoy what I eat … I suppose if a doctor turned round and said to me if you don’t change your diet you’ll be dead tomorrow, then I might change my diet. But it would have to be that drastic” *PC_14*

Perceived needs and requirements for self-management education and support	
*Desire to engage in self-management education and behaviours*	“I would find it very helpful if I had someone to sit and talk to me and explain. What situation my kidneys are at, what could happen, and how to look after myself. To keep my kidneys as, function as good as I can … And if I did need to change anything. I’d most definitely do that … It’s just making people more aware. I know some people find it harder than others to change their lifestyle. Because it can be a dramatic change, can’t it really” *PC_09* “Well I want know the symptoms and how to improve them. Or minimise them. … I would be very interested in that, yeah. I need to know how far down or how far up I am on the scale compared to what the norm is” *PC_12* “Would be good to know that what you can do to combat things … so gives you pointers of like losing weight or more healthy lifestyle, or things that will help you cope with it far better, as if and when it gets worse. So you know how to deal with it when it comes, when it happens” *SC_04* “I know nothing about kidney conditions. What I don’t know ain’t going to hurt me. Ignorance is bliss … If it became serious, that’s different … if anything went wrong with my kidneys, they’d soon tell me. Then I’ll deal with it” *PC_14*
*Perceived missed opportunity for early intervention*	“I think really right at the beginning, when you’re diagnosed. I mean it’s a bit late for me now, but as I say, it’s something that should be given at the time. I would know then what to expect” *PC_11* “Perhaps a leaflet on diagnosis, the fact that yeah, OK, so they’ve got to take on board they’ve got this condition, but they can go away then, when they’ve got a bit of time, to look at it when it’s all… When they’ve had time to just take on board what’s happening” *PC_01* “There’s not a lot about diet. This has been my real bugbear, I have to say. For people who are… For people who are let’s say pre-dialysis, dialysis, there’s quite a lot of information about diet, about what’s best to eat and what’s best not to eat. But I’ve found that there’s a real paucity of information if you’re let’s say between 30 and 40% kidney function. And I’ve had to kind of use my common sense” *PC_10*
*Format and delivery of self-management education and support*	“If advice could be given, in whatever form, either in written form or online or whatever, that would be good” *PC_07* “I’d say on a website, like that actually deals with that condition, rather than having one of those medical websites that deals with this condition, that condition” *SC_04* “If someone sent an email … it makes the information much easier to disseminate to more people than trying to print it. I mean the papers that I got, I printed them, I’d already lost them so – but I know where they are on the system” *PC_03* “I’m wondering how many people are out there that are kind of struggling, that have got nowhere to really turn to, apart from the GP who probably with the best will in the world may not be the best person to sort things out either for them. But somewhere where they can be proactive in their own care, if you like, and if that was on a website or something like that … where they can actually get that reassurance or they can get the information. Something like that, that guides them and that, because you’re not necessarily going to think of that stuff yourself” *SC_08*
*Provision from trusted sources*	“I would sooner read a pamphlet of the GP that is kosher information, as regards to looking online and looking on Dr Quacks… if the doctor turned round and said look, read this; this is going to help your kidneys out or your kidney condition out, and it’s a useful bit of information, you would read it, wouldn’t you? … You get it straight from the horse’s mouth then, as opposed to probably getting misinformation off somebody else, that’s not really a professional” *PC_05* “You’ve got to be careful about what information you access on the Internet and what books you read, because they might actually steer you in ways that aren’t necessarily that appropriate. So I think you’ve just got to think about what you’re reading, who is giving you this information” *PC_01* “If your GP or a nurse said to you, referred you to this. I think that would help me 100%, if I was referred to somebody, that you know… That would really help me, I’d find that a big help I think” *PC_09*

**Table 3:** Themes and subthemes of factors perceived to influence self-management

Knowledge and capability-related factors	
*Health literacy*	“Plain simple language, not these long medical words that I cannot pronounce, let alone understand … I had to spend hours and hours and hours trying to find decent sources of information out there” *PC_14* “I can’t remember, and it was probably technical jargon which I wouldn’t have understood anyway. So I probably, I tend to be like that, people tell me things, I get snippets and I forget the rest of the conversation” *SC_05*
*Perceived risk and controllability*	“It didn’t, didn’t over bother me to be quite honest with you. Because I thought I feel alright, so it can’t be that bad. And also I thought that if it was serious. Then they would be taking more action. Than they were doing, which was basically keeping a check on me” *PC_10* “Well, you can’t get wrapped up in these things can you? If it’s not hurting me, it’s not cause me huge amounts of problems” *PC_02* “There isn’t much you can do to be honest, is there? I mean if you’ve got a bad kidney, unless have like you say dialysis, that would be a bit worrying … but I don’t think I’m that far advanced” PC_06 “If I feel alright, that’s good enough for me. I know all the risks there is. I’m one of those people, I know my body better than other people do” *PC_11*
*Symptom burden*	“The tiredness, I just have to pace myself. I can’t go through the whole day being physically active without sitting down to some degree. And eventually the tiredness just gets to me, to the point where it affects my mental capacity. It’s quite overwhelming” *PC_01* “I get very out of breath. That is, and that persists even now I’m feeling better. If I was to walk quickly from now, here, to the car park, at what used to be my normal speed, I would have to sit in the car puffing and panting, you know. And then there’s the periods of tiredness, which are debilitating, but they fortunately only last a few days at the moment” *SC_01* “Tell you the truth, if my leg weren’t in pain, I would go and weed in me. Because me back’s nearly all slab…. I’d go and do a bit of weeding, but my legs won’t just stand it at the moment until I find out what I can take for it. I’m just been careful” *PC_08*

Skills and opportunity-related factors	
Psychological coping strategies	“Not worried too much about it, because whatever you get you get, and you’ve got to learn to accept it and just get on with it. So that’s what I’ve tried to do, but there are moments when you’re sitting there thinking about it” *SC_03* “The trauma of in a sense of finding out that I’ve got it, was huge, was massive. At the time. I’m not going to underestimate the impact. It was more psychological rather than anything else. Trying to get my head round it … I mean I did go through a period of time where I got quite depressed about it, and thought what’s the point?” *SC_08* “It would possibly do the opposite and make me more aware of the fact that I’ve got things wrong with me. I’d rather forget that there were things wrong with me … If it’s not hurting me and it’s not interfering why should I bother?” *PC_02* “At present to be honest I don’t, sort of developed a block and say everything is OK!” *SC_02*
Accessing and utilising health services	“You make an appointment to make an appointment, and I think oh, I’ll get better, so I don’t bother a lot, unless I think it’s something that needs following up …. If I haven’t got any symptoms, why worry myself?” *PC_13* “I go in, I see [Professor 1], come out, I feel great, because you’ve ticked the box again and you’re OK for three or four months. So I don’t need any more than that at the moment … you could spend a lot of time seeing different people, dieticians and whatever” *SC_05* “If I’d got any problems, I’d deal with them myself, because I don’t want to burden them. That’s probably why I’m not particularly worried about my ailments, that I don’t ask questions, because there’s nothing you can do about it” *SC_06* “I just say how is my kidney function doing after a blood test and they’ll say everything’s OK. You get, you ring up, you get your results. And it can be very hard to get to see your GP” *PC_14*
Forming and adapting routines	“I do alternate days: I’ll do cardiac one day and weights the other day. I might do a bit of swimming as well. The main reason I do that is because I have time … because I’m retired, I put myself for an hour a day, five days” *SC_07* “There’s different things, obviously there’s kidneys, there’s blood pressure, there’s the gout, so I’ve got a cocktail as it were … I’ll put them out for the morning … so they’re there when I’ve had breakfast I take those. So I don’t tend to forget. Because I’ve had them for so long, you just get used to knowing that you’ve got to take tablets … I suppose your lifestyle is your lifestyle, and then changing it, it depends how big the change is that you’ve got to do, and what you’ve got to do” *SC_05* “I’ve made my life much slower … there are all sorts of things like that that I used to do that I can’t do any more. But I’ve arranged my life so that I can manage what I attempt now. You know, I don’t do things that I know are going to be too much for me” *SC_01*
Self-regulating behaviours	“Yeah, I suppose if I can set myself a target, and just say right, by this time I’ve got to be doing so much a day, and give meself a [goal] … I just take each day as it comes. I just plod on with it” *SC_04* “I suppose as I achieved little things. It was slow, you just achieve a little bit at a time, and then suddenly you look back and you think goodness, I’ve got over that! … Obviously at the beginning it was very difficult … And looking back on it, I realise how much was achieved” *SC_01* “I spend an hour on the cross-trainer; I do a mile on that. That takes me about 18 min, I’ve got it down. And check my pulse rate. I keep the pulse rate down to about 100. I do my weights in between and then go on the treadmill for another mile … I have cut it down slightly, because my systolic blood pressure … I did an exercise on Sunday, and I’ve got a monitor at home, and I routinely every month or so check my blood pressure. And the systolic blood pressure was a bit high” *PC_12*

Confidence and motivational-related factors	
Self-efficacy	“I wouldn’t say I’m confident in myself. There are things that I don’t feel confident with, but I don’t know, I just take each day as it comes and just go on as it is. I’ve got no problems that way” *SC_04* “I used to swim a lot, actually … But because I’m feeling a bit pathetic at the moment because. I mean at least walking I can stop; if I get slightly breathless I can stop. At the moment I feel a bit self-conscious about it, in water, if there’s lots of other people around kind of thing. It might sound a bit silly, but no. I’d be OK if it was kind of leisurely and there were not too many people around, if I’m in the pool, but I don’t want to be getting too breathless when I’m halfway down a length. I was a really really good swimmer at one point” *SC_08* “There’s a centre there, exercise centre … And I went a couple of times, and I thought I can’t do this. There’s no way I can stand in front of a mirror, look at myself and lift weights and row a boat, it just doesn’t appeal to me at all. You know, you’ve got the other men there all doing this in the mirror, I’m sorry, it weren’t for me so I didn’t go any more” *SC_06*
Willingness to change	“If somebody tells me to do something and I don’t want to do it, I just, I just will not. You can cajole, you can do anything, but if I will not. If I don’t want to do it I just won’t do it. So I’m the one that’s got to give myself the metaphorical kick up the backside and do it. So I’m the one that’s got to do it myself. Other people can suggest it, and have a go at me, like me other half, but at the end of the day I’m the one that’s got to physically do it myself. There’s not much I can think that can motivate me to do it. It’s just got to be me” *SC_04* “I’m overweight, I’m well aware of that, and the readings that they showed me are a bit shocking, I must admit. But yeah, it’s been mentioned. I have to say, I’ve probably not, I’ve not taken them on board if truth be told. Probably because I enjoy the lifestyle I have … I always have done. It would be unfair to say I don’t feel the need to, because I probably do, if it’s killing me … But I feel well enough and I’m happy with what I do. So I’ve not got that real incentive, I guess, to make those changes” *PC_10* “I feel listless and I can’t be bothered…. Because I can’t do it in the way I used to, I’ve lost a bit of motivation. When I knew I could go and swim 40 lengths straight off, feel much better. Now I go and do 10 and I’m struggling, it’s not so exciting, is it!” *SC_01* “I think I’ve always had that mindset that if you look after yourself it’s worthwhile doing. And I think that being a nurse you value it even more. With a nursing background you can see what happens to people who don’t look after themselves. There’s nothing that would prevent me from doing my best. I feel motivated. I can see the value” *PC_01*
Changes in health status	“Obviously if you get something the matter with you then it’s going to probably prevent you doing something or other, or you don’t feel quite as well” *PC_04* “It’s limiting. I wouldn’t say, it doesn’t stop me completely, no. It’s not got to the point where it’s stopping me. Because I’m quite determined to do stuff anyway, and to keep independent. I think once you start giving up then you’re on an even… More of a speedy downward trajectory…. I’m trying to do as much as I can to help myself at the minute” *SC_08* “Other than the aches and pains, which I am following up. I’ve got two physio appointments, and they’ll maybe do something for me. I’m trying to do things myself … I want to be around as long as I can and enjoy it. Once I don’t enjoy it I want to go… I don’t want to linger. I visit people in homes, and it’s not life, it’s existing” *PC_13* “I don’t want to go through being cared for. So one of the things I do is actually say one way I can make sure, with god willing, is to stay healthy” *SC_07*

Social support	
Familial support	“I’d got my husband there all the time. I mean that was helped by us both being retired as well, because he was there. It would have been very different if he had gone out to work and left me all day. So somebody who was affected much younger, because I don’t know how I would have managed if I’d got children at home or anything. I mean I think those people would need a lot more support” *SC_01* “But I am independent person, and if sort of… If I’m feeling unwell, I won’t tell anybody or say anything. I just keep it to myself. You know?.. I don’t know. I suppose I don’t want to bother anyone by saying I’m not feeling good today, you know” *PC_09* “I’m not so independent as I was, and it. [Mutters] me off. Yeah, really does. I’ve got to rely on other people … and I don’t like it … when you live on your own, there’s not a lot of activity you can do!” *PC_14* “Couldn’t ask your wife or the son about it, could you? I mean what would they know about it? I don’t think anybody knows what your kidney does” *PC_06*
Peer support	“When you’re talking to somebody else that’s got the problem, I can’t see the point in it, because everybody, not person, but body, everybody is different … To talk to, yes, tell each other the different tales of what’s wrong, but I don’t think one’s going to help the other” *SC_06* “I would love to do, because you can kind of shared experiences. Whenever I’ve been in hospital, it’s the same if you’re in a ward and you’re all there for similar stuff, and you do, you do talk. And you know, it is kind of supportive in a way, isn’t it? … People are not necessarily going to have the support network that they need. Or maybe they do have the support network but it’s not necessarily what they need either” *SC_08* “It would be helpful because then, I mean they can tell you what their kinds of symptoms are, and you might think of oh, well I have that symptom. So it is good to chat to other people with it … you would learn from each other. And it’s that support, if you’re going through similar circumstances to what they are, it’s nice to know there’s other people out there in your situation that you can chat to about it” *PC_09*
Healthcare professional support	“I am getting the support now every three months I go, four months, I see [Professor 1]. It’s nice to go and have a chat with him. It’s nice to know that support is the fact that they have taken the readings, everything seems to be OK, blood pressure is fine, and you get a tick of good health. And that’s the support that keeps me” *SC_05* “I’ve got a brilliant doctor. Doctor is absolutely wonderful, [Doctor 2], I’ve never known a doctor like him. I could talk to him if I wanted to, again. I’m conscious that he is very busy. He doesn’t want to be bothered with someone like me” *SC_06* “I saw the consultant again, and I had to tell her, can I reduce the dosage? She kind of like said ‘well if you want, reduce it to this’. I wish she could have said to me right, here is the results: here’s the result of the last two, three, four, six months. Because I did ask, can you decide for me? She said no, I can’t decide. I don’t know if she has taken the safe option or something” *SC_07* “If I think I’m going off for any reason, I will get myself seen to … My GP is very good at knowing when I need referring for something doing or not. The Surgery as a whole is very good for doing routine stuff, ‘it is about time you had this done, that done, the other done’” *PC_02*

### Perceptions and experiences of (self-)managing CKD

#### Lack of awareness and understanding of CKD and its self-management

There was a lack of awareness and understanding of CKD, what self-management is or how it could be defined. Many believed that there was not much that they could do to help manage their kidney health. CKD management was thought to only include medical management, including attending appointments, taking medication and engaging with their healthcare provider(s). Many participants were unaware of other aspects of self-management, such as behaviour (e.g. diet, physical activity) and emotional (e.g. coping).

#### A paucity of information received

Participants described receiving very little or no information regarding their CKD and its management. Whilst many understood that they had reduced kidney function, they did not comprehend what this actually meant and the potential consequences. Participants expressed a desire for information about their CKD, what they might expect with regard to disease progression, and how best to look after their health. Whilst it was believed that there should be information available, participants thought that their doctor would give them the information if they required it.

#### Downplay of kidney disease

Participants discussed how they perceived that their CKD was not a significant issue or problem given the lack of concern expressed by their doctor. Consequently, this led them to believe that they did not need to worry about their condition or engage in CKD self-management strategies or behaviours. The apparent nonchalance of their doctors resulted in participants feeling reassured about their CKD, and thus, the majority were not overly concerned about the potential health risks or complications associated with CKD; conversely, this made other participants more anxious.

#### Engagement with self-management behaviours

The perception that their CKD was not hurting or interfering with them resulted in some participants dissociating from engagement in health-promoting behaviours as strategies to better (self-)manage their condition. Whilst many participants were engaging in behaviours synonymous with a healthy lifestyle, they were unaware that these behaviours were important for CKD self-management. The dearth of information available for those with earlier stages of the disease, especially lifestyle information, left participants feeling like they had to use their judgment.

Perceived needs and requirements for self-management education and support.

#### Desire to engage in self-management behaviours

There was a desire to learn more about CKD self-management, its importance and self-management strategies. Participants wanted to know how self-management behaviours could affect their CKD, particularly the impact on kidney function and disease progression. Several participants were eager and willing to take action to better self-manage their CKD but lacked knowledge and confidence about other self-management strategies beyond pharmacotherapy. Some participants were not interested in engaging in self-management despite the potential consequences.

#### Perceived missed opportunity for early intervention

Participants believed that the necessary information and support to help people self-manage their condition should be provided at earlier stages of the disease, as close to diagnosis as was appropriate. It was highlighted that most CKD literature is targeted towards those who are nearing kidney failure (CKD stage 5), or those receiving kidney replacement therapy. It was perceived that information and support were provided when it was almost too late to do anything, and not when it could potentially have the greatest impact.

#### Format and delivery of self-management education and support

The need for self-management information and support was evident, with many participants advocating for it. Participants acknowledged that variations exist between individuals in terms of people’s abilities to understand information, capabilities, opportunities, needs and priorities, and thus self-management information and support should be individually tailored. Whilst most participants preferred information online, ideally a dedicated kidney-specific website, some wanted printed information.

#### Provision from trusted sources

Receiving information from trusted sources was considered important and influential in health decisions. Participants reported trusting information received from, or signposted to, by their doctor or another known healthcare professional rather than information that they found online themselves. Some were dubious or overly cautious about information found online, especially from non-UK sources.

### Knowledge and capability-related factors

#### Health literacy

Participants who accessed or received information about CKD found it difficult to understand and process the information, largely due to the complexity of information and use of medical jargon, making it hard to navigate and decipher. It was challenging for participants to determine the trustworthiness and reliability of information found online. Some participants described suffering from misinformation as a result of believing and following fictitious information that contradicted advice from healthcare professionals.

#### Perceived risk and controllability

Many participants perceived that their CKD was not affecting them, and they did not need to take action as a result to prevent exacerbation or worsening of their CKD. Some participants considered CKD to be uncontrollable, with little that they could do to improve or manage their CKD. Those who perceived their CKD as a threat were more likely to engage in self-management behaviours, particularly those that would reduce potential health risks.

#### Symptom burden

Symptom burden was considered to impair individuals’ abilities to perform functional activities; some activities were more challenging while others were no longer possible. Activity management, including knowing one’s own limits, was frequently reported with individuals using strategies, like pacing, adjusting, or taking regular breaks, to ensure completion of tasks. Symptoms were perceived to be an indicator of health status, with many using changes in symptoms as a reason to seek treatment.

### Skills and opportunity-related factors

#### Psychological coping strategies

Maintaining good mental health and wellbeing was perceived to be vital to cope with the physical, psychological and social impact associated with CKD. Most were accepting of their CKD diagnosis and potential disease trajectory; however, some participants preferred not to know. Whilst acceptance appeared to result in more effective psychological and emotional self-management, this was not always apparent for physical self-management. Avoidance was used by several participants as a coping mechanism to prevent them from feeling overwhelmed.

#### Accessing and utilising health services

Participants felt that there was limited discussion about their condition and appropriate self-management, and thus it was perceived to be unimportant. Many participants were neither confident nor willing to ask questions during appointments for fear of wasting healthcare professionals’ time. Participants reported not seeking further information and support as they were unaware of what was available and how to access it. Being informed about test results and what they meant, alongside advice about how to act in response was considered important for self-management.

#### Forming and adapting routines

Building a daily routine and having regularity was believed to assist with behavioural maintenance. For many participants, their routines were well-established prior to their CKD diagnosis, especially for those with pre-existing health conditions. Increasing age, increased symptom severity, and decreased physical function resulted in participants adapting their routines to help them better manage. Adopting new self-management strategies or health behaviours was considered to be challenging.

#### Self-regulating behaviours

Creating and setting health-related goals was considered to provide motivation to improve health behaviours. Achieving goals resulted in feelings of satisfaction and accomplishment, and a desire to continue the new behaviours and setting of new goals. Self-monitoring of activities, particularly those associated with the medical aspects of self-management, like monitoring blood pressure, reviewing blood test results, and managing medications, were frequently reported.

### Confidence and motivational-related factors

#### Self-efficacy

Participants perceived that they were unable to take greater control or responsibility for their own health as they were not confident in their abilities to perform self-management behaviours; this was mostly as a result of not knowing how to effectively engage and implement self-management strategies into their daily lives. Experiencing improvements in their health left participants feeling empowered with an increased self-belief in their ability to make and sustain further changes to improve their health.

#### Willingness to change

There was a lack of motivation and desire for participants to change their lifestyle behaviours due to many being content with their existing lifestyle. Changes in participants’ ability to participate in health-promoting behaviours, like physical activity, influenced their levels of motivation and willingness to adopt new and/or adjust current health behaviours. Increased self-motivation and willingness to change were considered key factors to improve engagement in effective self-management behaviours.

#### Changes in health status

Many participants were concerned about deteriorations in their health which may impact their ability to participate in functional life activities. Several participants described feeling a sense of responsibility and receptiveness to look after their own health to enable them to be as healthy as possible for as long as possible. Maintaining good health status was a motivating factor for participants to ensure preservation of their health enabling them to remain independent without feeling like they were reliant, or a burden, on their relatives.

### Social support

#### Familial support

Support from relatives was believed to play an important and influential role on the participants’ health and lifestyle, helping them to better cope with and manage their condition. Most participants discussed how they relied on their support network to help them manage day-to-day activities. However, a small number stated that they had not disclosed their CKD to their relatives. Participants discussed the difficulties in accepting help and support from their relatives and feelings of guilt and fear of burdening them.

#### Peer support

There were conflicting opinions about the benefits of support from others living with CKD. Many expressed a desire to share experiences and learn from others with similar circumstances to enable comparisons; it was believed that this could help participants understand their condition better, how they can best manage their CKD, and what successful self-management may look like. Direct comparison to peers was viewed as intimidating and had the potential to deter or discourage participants from participating or replicating desired behaviours.

#### Healthcare professional support

The majority of participants believed that they had a good relationship with their doctor and healthcare team, and felt well-supported. Having regular appointments provided reassurance and an opportunity to discuss any issues they had, although participants were conscious of the limited time available. A couple of participants discussed making shared decisions with their doctor, mainly around medications. Healthcare professionals were considered to be influential by participants adopting and engaging in self-management strategies.

## Discussion

This study describes the experiences and perceived requirements for self-management information and support, as well as the barriers and facilitators to self-management for people with non-dialysis CKD from the UK. This information is important for understanding how to support people in improving their self-management behaviours. The findings highlighted that people with CKD lack awareness and understanding of CKD self-management, but do have a desire to learn more about how they can better manage their CKD, adopt self-management strategies, and engage in health-promoting behaviours. Our findings demonstrate that several influential factors need to be considered when developing and implementing self-management education and support resources.

Discussions with healthcare professionals about CKD and its management were reported by participants to be non-existent or minimal, despite a desire to learn more and a willingness to take action. Limited communication regarding self-management has previously been identified, particularly around the importance of self-management for CKD [14]. Participants believed that their CKD was not a significant issue or problem. These perceptions may be because other health conditions, such as hypertension and diabetes, have a higher profile and are often prioritised during clinical appointments [15], but may also be because of the lack of concern expressed by doctors about the significance or implications of a diagnosis of CKD. The lack of understanding about disease progression and the potential complications of CKD made it difficult for participants in this study to comprehend the consequences of poor self-management. This highlights the need for healthcare professionals to clearly communicate the seriousness of CKD in a way that is informative but not alarming, emphasising its progressive nature and the benefits of early intervention. Normalising discussions around CKD and addressing both informational and emotional needs can help improve awareness and understanding. Learning the beneficial effects of self-management can result in benefit-based motivation (e.g., better blood pressure control or improved physical fitness) or consequence-based motivation (e.g., slowing CKD progression, avoiding dialysis), which can ultimately influence behavioural performance and maintenance [14]. Engagement in CKD self-management strategies and adherence to relevant health-promoting behaviours (e.g. maintaining a specific diet, and monitoring fluid intake) are often driven by the belief that these actions will prevent CKD progression [16].

People living with CKD often lack awareness of the condition and the detrimental effect poor risk factor control (e.g. diabetes and hypertension) may have on their health [17]; thus, it is unsurprising that participants in our study perceived low-risk perceptions of CKD. Like Lissanu et al. [18], participants were unaware of the severity of their CKD or what might happen if their CKD progressed, or their kidneys failed. Lack of knowledge and understanding may adversely influence an individual's ability and motivation to engage in self-management behaviours to prevent the progression of CKD [18]. Alternatively, patients may be in denial about the potential health risk factors, or this may serve as a coping mechanism in response to changes in health status. For people with CKD, denial may help in the short-term to cope with the stress associated with their CKD diagnosis and prognosis but may be unfavourable in the long-term when it prevents individuals from preparing for disease progression or kidney failure [18]. Individuals with lower levels of knowledge, skills, and confidence to look after their health are at risk of engaging in more avoidant coping strategies [19]. Addressing individual barriers, such as low health literacy and lack of confidence, and supporting appropriate coping strategies to live with and manage their condition is imperative to improve patient activation for those living with CKD. Targeted interventions and support designed to increase patient activation could potentially improve the perceptions of CKD and associated risk factors and encourage engagement in adaptive self-management strategies.

Several barriers and facilitators were identified which influenced participants’ engagement in self-management behaviours. Having the knowledge and ability to access health information and health services, and partnerships with healthcare professionals to improve and maintain health actions and behaviours, known as health literacy [20], was believed by participants to affect self-management behaviours. Health literacy is a vital determinant of effective self-management behaviour [21, 22], with limited health literacy associated with poor CKD self-management behaviours, such as adherence to medications, fluid and dietary restrictions and lifestyle modifications [22]. Whilst those with earlier stages of CKD have been found to have better health literacy compared to those with more advanced disease, they have the worst self-management behaviours [21] that may be explained by the asymptomatic nature of early stages of CKD where there is a lack of awareness and it is easier to ignore the condition [23]. Self-efficacy and empowerment have been identified as mediators in the relationship between health literacy and self-management behaviours across various long-term conditions including CKD [24]. Providing the necessary health information and support, alongside building confidence, can help empower individuals to take a more active role in their health and manage their kidney disease [7], and thus should be incorporated into self-management care and interventions.

Routinising behaviours and setting goals were considered to facilitate consistent engagement in self-management behaviours. These findings are similar to other studies exploring barriers and facilitators to CKD self-management [14, 25]. Schrauben and colleagues [14], who also identified barriers and facilitators to CKD self-management, proposed that self-regulation theory can be used to determine where patients with CKD are at in terms of engagement in self-management behaviours and to guide discussions between patients and healthcare professionals to optimise patients’ ability to proceed to the next phase and/or maintain the behaviour. Promoting self-regulation and adherence to behaviours, such as self-monitoring, can support consistency and maintenance of self-management [14, 26]. Additionally, like others [14, 25], we found that social support networks were perceived to predominantly be beneficial for encouraging people with CKD to self-manage. Social support plays a dominant role in the self-management behaviours of people with CKD [21] by offering valuable support and accountability that is fundamental to successful behaviour change [27]. Self-management interventions should incorporate elements of social support to encourage people to improve their health and lifestyle behaviours. Involving people living with CKD in the development of educational resources and interventions can enhance their relevance and impact, and facilitate meaningful health behaviour change.

Whilst variations existed amongst participants about perceived requirements and delivery of CKD self-management education and support, the desire to learn more and act on the information was evident. Digital health interventions may provide a potential mechanism to access health information and services for people living with CKD [28], by addressing several implementation barriers often associated with face-to-face interventions, such as time and transport. Accessing health information online is increasingly common but can result in inconsistent information or misinformation if searching on irrelevant sites, something participants in our study and other studies [29] were concerned about. Digital resources need to be provided, or signposted to, by healthcare professionals to guide patients to trustworthy and accurate online health information and to support safe health-seeking behaviours [30]. Ensuring that the health information provided is what patients want and perceive they need is key for successful behaviour change. A recent qualitative systematic review with meta-ethnography of the preferences of people with chronic kidney disease regarding digital health interventions that promote healthy lifestyles identified important considerations to ensure that future interventions are tailored to specific needs and goals [29]; patient preferences included simple instruction and engaging design, individualised interventions, virtual communities of care, education and action plans, timely reminders and automated behavioural monitoring [29]. The findings from this study align with those from the meta-ethnography, with the identification of the needs and barriers, alongside the priorities of people living with CKD. Both highlight the importance of simple and engaging guidance, personalised support, health-promoting education, behaviour change support strategies, and monitoring. Consideration of patient preferences could support the effective design and development of digital health interventions for people with CKD, which are gaining increasing recognition. Involving individuals in the development of digital health interventions can ensure that they meet their needs and priorities, and are user-friendly, which will facilitate better engagement and improved outcomes.

The main limitation of this qualitative sub-study is that it was conducted at a single location at a single time point. Thus, the findings may not be generalisable to the wider CKD population. In addition, self-management behaviours are dynamic and dependent on several factors that may respond or react differently according to the circumstances. Despite using purposive sampling to capture a diverse range of participants, our study population was largely homogeneous, with the majority of participants identifying as White British. Most previous qualitative studies on self-management have focused on more advanced stages of CKD, those receiving dialysis, or transplant recipients; thus, the rich data collected in this study adds new knowledge to the limited literature on self-management in non-dialysis CKD. In addition, the findings of this study have contributed to the co-development of a self-management digital health intervention, called My Kidneys & Me [31], which has demonstrated positive effects on patient activation and self-management behaviours in people living with CKD [32]. Whilst My Kidneys & Me has been well-received and used by participants [33], further work is required to improve the equity for people from disadvantaged or underserved groups. Specific revisions to improve accessibility and engagement for these populations will be undertaken.

## Conclusion

This study has highlighted the lack of patient awareness and understanding of CKD and its management, in conjunction with a real desire to learn and action appropriate self-management strategies and behaviours. The identification of perceived barriers and facilitators to participation and engagement in health-promoting behaviours can be used and incorporated in the development of interventions to support self-management in people living with CKD. Effective resources to improve patients’ knowledge, skills, and confidence in managing their CKD, including associated health risks, are needed, and successful implementation of such interventions warrants attention.

## Supplementary Information

Below is the link to the electronic supplementary material.

Supplementary file1 (DOCX 21 kb)

## Data Availability

The data that support the findings of this study are available from the corresponding author upon reasonable request.
