# Supplementary material for: Supporting people with chronic kidney disease to self-manage their condition: understanding the lived experiences, needs and requirements, and barriers and facilitators
Source: J Nephrol. 2025 Jul 12;38(7):1969–84. doi: 10.1007/s40620-025-02349-8 (PMC12484093; doi:10.1007/s40620-025-02349-8)
Supplement: Supplementary file 1 — Supplementary file1 (DOCX 21 kb) [file 40620_2025_2349_MOESM1_ESM.docx]

Supplementary references

S1. Malterud K, Siersma VD, Guassora AD. Sample Size in Qualitative Interview Studies: Guided by Information Power. Qualitative health research. 2016;26(13):1753-60. doi: 10.1177/1049732315617444.

S2. Corbin JM, Strauss A. Unending work and care: managing chronic illness at home. San Francisco: Jossey-Bass; 1988.

S3. Tracy SJ. Qualitative Quality: Eight “Big-Tent” Criteria for Excellent Qualitative Research. Qualitative Inquiry. 2010;16(10):837-51. doi: 10.1177/1077800410383121.

S4. Braun V, Clarke V. Reflecting on reflexive thematic analysis. Qualitative Research in Sport, Exercise and Health. 2019;11(4):589-97. doi: 10.1080/2159676X.2019.1628806.
